# Immunosuppressant nonadherence profile in kidney transplant recipients and the impact of medication adherence on transplant outcomes

**DOI:** 10.3389/fphar.2024.1493166

**Published:** 2024-12-18

**Authors:** Zou Zhi-yu, Dai Lin-rui, Yu Chen-zhen, Chen Ren-jie, Yu Fei-hong, Chen Song, Chang Sheng, Zhang Wei-jie

**Affiliations:** ^1^ Institute of Organ Transplantation, Tongji Hospital, Tongji Medical College, Huazhong University of Science and Technology, Key Laboratory of Organ Transplantation, Ministry of Education, NHC Key Laboratory of Organ Transplantation, Key Laboratory of Organ Transplantation, Chinese Academy of Medical Sciences, Wuhan, China; ^2^ Kidney Transplant Department, Organ Transplant Center, Third People’s Hospital of Shenzhen, The Second Affiliated Hospital, Southern University of Science and Technology, National Clinical Research Center for Infectious Disease, Shenzhen, China

**Keywords:** kidney transplantation, immunosuppressant, nonadherence, rejection, graft outcomes

## Abstract

**Background:**

Despite the fact that 1-year graft and recipient survival rates are above 90% in most transplant centers, improving long-term graft survival remains an important challenge. Immunosuppressant nonadherence has been recognized as one of the important risk factors for long-term graft failure. Understanding the modifiable correlates and risk factors for medication non-adherence is essential to develop interventions to improve adherence and thus long-term transplantation outcomes.

**Methods:**

This study conducted a questionnaire survey on 431 renal transplant recipients who were followed up in the outpatient clinic between January 2022 and January 2023, and 409 valid questionnaires were returned. The BAASIS questionnaire was used to assess the prevalence of nonadherence to immunosuppressive therapy (implementation phase) in Chinese renal transplant recipients and to explore the multilevel correlates of immunosuppressive nonadherence. The BAASIS questionnaire was used to categorize renal transplant recipients into adherent (n = 239) and non-adherent (n = 170) groups, and a prospective cohort study with a 1-year follow-up was conducted to explore the impact of immunosuppressant non-adherence on clinical outcomes.

**Results:**

The prevalence of nonadherence to immunosuppressant therapy in renal transplant recipients in this study was as high as 41.6%. The number of years post-transplant (OR: 1.240, 95% CI: 1.136–1.353, *p* < 0.001) and the frequency of twice-daily dosing (OR: 5.145, 95% CI: 2.690–9.840, *p* < 0.001) were positively correlated with immunosuppressive nonadherence. There was a significant difference in TAC IPV (Intra-individual Variability) between the adherent and nonadherent groups (22.7 ± 8.7 vs. 25.4 ± 11.6, *p* = 0.010). Renal function remained stable during the follow-up period in the recipients in the adherence group and tended to decrease in the recipients in the non-adherence group (F = 4.932, *p* = 0.001). The rates of graft loss (7.1% vs. 1.7%, *p* = 0.006) and rejection (12.4% vs. 4.2%, *p* = 0.002) were higher in the nonadherent group than in the adherent group.

**Conclusion:**

Longer time post-transplant and higher frequency of immunosuppressive dosing were positively associated with nonadherence to immunosuppressives medication. Immunosuppressant nonadherence was associated with adverse graft outcomes.

## 1 Introduction

Kidney transplantation (KT) is a primary treatment for end-stage renal diseases. Although progress has been made in short-term outcomes of KT, the loss of long-term allografts remains a key challenge (Lentine et al., 2022). The COMMIT (Clinical Checklist by the Consensus on Managing Modifiable Risk in Transplantation) consensus identifies modifiable factors for low long-term survival in renal transplant recipients as poor adherence, high intra-individual variability (IPV) in immunosuppressant trough concentrations, inadequate/excessive immunosuppression minimization, immunosuppression-associated adverse reactions, *de novo* donor-specific antibodies (dnDSA), early ischemia/reperfusion injury, delayed recovery of graft function (DGF), cardiovascular and metabolic complications. Of these, poor adherence is considered to be an independent risk factor for poor prognosis in renal transplantation (Neuberger et al., 2017). Nonadherence to immunosuppressive medication has long been considered a modifiable risk factor for long-term graft failure (Sanders-Pinheiro et al., 2021; Villeneuve et al., 2020; Gandolfini et al., 2022). Medication nonadherence increases the intrapatient variability (IPV) of immunosuppressive medication concentrations, which may lead to the development of *de novo* donor-specific human leukocyte antigen antibody (dnDSA) in recipients and increases the risk of long-term graft rejection and loss (Rodrigo et al., 2016; Sapir-Pichhadze et al., 2014; Cherukuri et al., 2019). Reports indicate that 15%–60% of late acute rejections and 35%–45% of graft losses are associated with medication nonadherence. Therefore, understanding the modifiable correlates and risk factors for medication nonadherence is critical for developing interventions to improve adherence and thus long-term transplant outcomes (Sanders-Pinheiro et al., 2021; Nevins et al., 2017).

The Kidney Disease: Improving Global Outcomes (KDIGO) Transplant Work Group identifies nonadherence as “deviation from the prescribed medication regimen sufficient to adversely influence the regimen’s intended effect” (10). As defined by the Ascertaining Barriers to Compliance (ABC) taxonomy, medication adherence refers to the process by which patients take their medications as prescribed, and is further categorized into three quantifiable phases: “initiation,” “implementation,” and “discontinuance” (Vrijens et al., 2012). ABC has also reported rates of nonadherence to medication as high as 50% in developed countries and even higher in developing countries (Kidney Disease: Improving Global Outcomes KDIGO Transplant Work Group, 2009). A review of studies showed a nonadherence prevalence rate ranging from 36%–55% in KT, higher than that for other solid organ transplant recipients (range, 7%–15%) (Dew et al., 2007; Gustavsen et al., 2019; Schäfer-Keller et al., 2008; Gokoel et al., 2020). This variability is likely due to the use of different diagnostic methods with different specificities and sensitivities (Gokoel et al., 2020). Adherence can be assessed using direct or indirect methods (Villeneuve et al., 2020; Gokoel et al., 2020). Direct methods are designed to directly measure a patient’s drug intake and include directly observed therapy, radio-observed therapy, and therapeutic drug monitoring (Gandolfini et al., 2022). Directly observed therapy is the administration of medication supervised by a healthcare professional or caregiver, which is time-consuming, costly and not easy to implement in the clinic. Wireless observational therapy (WOT) based on an ingestible sensor system embedded in a pill or capsule can theoretically determine 100% of the actual amount and duration of drug intake to assess patient medication adherence, but may suffer from gastrointestinal adverse effects, monitoring of anxiety, and other adverse effects (Eisenberger et al., 2013). In addition, the high cost of WOT limits its widespread use. Therapeutic drug monitoring is used to directly assess drug intake, and adherence is usually assessed using tacrolimus trough concentration intra-individual variability [IPV], which can be expressed by calculating the drug level variation index (MLVI), standard deviation (Tac SD), coefficient of variation (CV), and Tac dose-concentration ratio (Shemesh et al., 2017; Shuker et al., 2015a; Whalen et al., 2017; Leino et al., 2019). Indirect methods include pill counting, electronic monitoring and self-reporting questionnaires. Electronic monitoring is based on the use of expensive microprocessors that are embedded in drug containers or blisters to record the time and date of drug intake, and is also limited by its high cost, which discourages widespread use (Lam and Fresco, 2015). Self-report questionnaires are cheaper, more convenient and easier to administer for assessing adherence, with the Basel Assessment of Adherence to Immunosuppressive Medications (BAASIS) questionnaire being the most commonly used, validated and widely used in kidney transplantation (Gandolfini et al., 2022; Gokoel et al., 2020; The Basel Assessment of Adherence; Basel Assessment of Adherence to; Denhaerynck et al., 2023). The rest of the questionnaires including the Immunosuppressive Therapeutic Adherence Scale (ITAS), the Simplified Medication Adherence Questionnaire (SMAQ), the Identification of Medication Adherence Barriers Questionnaire (IMAB-Q), and other validated self-report questionnaires. Although questionnaires may underestimate nonadherence in recipients, they can serve as an initial screen for nonadherence (Neuberger et al., 2017).

Medication nonadherence of kidney transplant recipients is influenced by several factors. The World Health Organization has defined five major categories of risk factors that may influence adherence behavior: social- and economic-related factors, health system/healthcare team-related factors, therapy-related factors, condition-related factors, and patient-related factors (Burkhart and Sabaté, 2003). Studies on the multilevel correlates of immunosuppressive nonadherence after heart and renal transplantation, including BRIGHT (26–28) and STICK(3) BRAZIL, have been guided by Bronfenbrenner’s ecological model, which assumes that individual behavior is the result of multilevel determinants, with the patient in the center (patient-level), followed by the influence of their healthcare provider/family (micro-level), the healthcare organization (meso-level), and the healthcare system and related policies (macro-level) (Berben et al., 2012). The BRIGHT study included 1,680 heart transplant recipients from four continents, 11 countries, and 36 centers, and explored multilevel factors associated with nonadherence to immunosuppressive medications in heart transplant recipients, making it the largest adherence-related study of solid organ transplantation to date (Denhaerynck et al., 2018; Schönfeld et al., 2020; Marston et al., 2023). The ADHERE BRAZIL, a multicenter, cross-sectional study in Brazil, investigated adherence in 1,105 kidney transplant recipients from 20 transplant centers in Brazil, exploring multilevel factors associated with immunosuppressant nonadherence in the kidney transplant population (Sanders-Pinheiro et al., 2021). A recent Ethiopian single-center cross-sectional study similarly explored the level of adherence to immunosuppressant medications and associated factors in renal transplant recipients (Derejie et al., 2024).

Due to the differences in ethnicity, culture, and healthcare among different countries, it is worthwhile to explore the current status of immunosuppressant nonadherence in the Chinese renal transplantation population, and it is hoped that appropriate interventions to improve adherence can be found by exploring the risk factors associated with nonadherence. Few reported studies exist on adherence in Chinese renal transplant recipients, and information on the correlation of IPV with nonadherence is limited (Teng et al., 2015; Zhao et al., 2017; Chen et al., 2022; Yang et al., 2022). Therefore, the aims of this study were to assess the prevalence of nonadherence to immunosuppressive therapy (implementation phase) in Chinese renal transplant recipients, to explore the multilevel correlates associated with nonadherence, and to investigate the impact of nonadherence on transplantation outcomes.

## 2 Materials and methods

### 2.1 Study area and period

This study was conducted at the Institute of Organ Transplantation, Tongji Hospital, Tongji Medical College, Huazhong University of Science and Technology, China. There were 84 beds in the clinical department of our transplant center. Our transplant center has been at the forefront in China in terms of three comprehensive indexes of transplantation programs, cumulative number of cases, and long-term survival. It is the first unit in China to have performed over 7,000 renal transplants, which represents the national lead in this category, providing a relevant environment for our research on adherence in renal transplant recipients. The study period is from January 2022 to January 2024.

### 2.2 Design and study population

#### 2.2.1 Patients

This was a prospective cohort study. This study included kidney transplant recipients who were followed-up in the outpatient clinic between January 2022 and January 2023. The main inclusion criteria were as follows: (i) age >18 years, (ii) >1-month post-transplant, (iii) treatment with immunosuppressive drugs containing Tac at follow-up, and (iv) the ability to understand the objectives of the study and provide written informed consent. The major exclusion criteria were pediatric recipients aged <18 years, treatment with immunosuppressive drugs not containing Tac at follow-up, and multi-organ transplant recipients. A total of 409 validated adherence assessment questionnaires were collected from kidney transplant recipients who met the inclusion and exclusion criteria. The incidence of nonadherence to immunosuppressive therapy (implementation phase) was calculated. Patient-level (sociodemographic, clinical, and treatment-related factors) predominantly, as well as micro-level (family/healthcare provider), meso-level (transplant center), and macro-level (healthcare system) variables were collected to analyze the multilevel correlates of nonadherence in kidney transplant recipients. Kidney transplant recipients were categorized into adherent and non-adherent groups based on the BAASIS interview version of the adherence assessment questionnaire. The baseline of follow-up was at the time of adherence assessment, and the follow-up data of kidney transplant recipients were collected for 12 months thereafter to investigate the effect of immunosuppressive nonadherence on transplantation outcomes.

#### 2.2.2 Immunosuppression

All patients who underwent KT surgery were treated with basiliximab or thymoglobulin for immunosuppressive induction therapy. All patients were treated with a standard triple TAC-based immunosuppressive regimen including MPA and steroids. Immediate-release tacrolimus (Prograf^®^, Astellas Ireland Co. Ltd., IR-TAC) was administered orally twice daily and extended-release tacrolimus (Advagraf^®^, Astellas Ireland Co. Ltd., ER-TAC) was administered orally once daily, with target tacrolimus blood levels of 7–10 ng/mL in the first year postoperatively and 6–8 ng/mL thereafter. TAC trough levels are measured at each clinic visit and the dose is adjusted to keep trough levels within the target range. The MPA oral dose is 500–750 mg, (or 360–540 mg of enteric mycophenolate mofetil) taken orally twice daily. The dose of prednisone acetate tablets was maintained at 5–10 mg once daily.

This study was conducted in accordance with the principles of the Declaration of Helsinki. The study protocol was registered with the China Clinical Trial Registry (ChiCTR2200061089) and approved by the Ethics Committee of Tongji Hospital, Tongji Medical College, Huazhong University of Science and Technology (TJ-IRB20220618).

### 2.3 Variables and measurements

#### 2.3.1 Adherence to immunosuppressant therapy

The validated interview version of BAASIS was used to assess the implementation stage of adherence to immunosuppressant therapy (with permission) (Sanders-Pinheiro et al., 2018; van Zanten et al., 2021). This self-report questionnaire is consistent with the ABC classification of medication adherence and uses four items (taking and timing adherence, drug holidays, and dose reduction) to measure the implementation phase of nonadherence to immunosuppressants (implementation nonadherence). KT subjects who reported deviations in any item within the past 4 weeks were considered nonadherent. The concurrent and predictive validity of BAASIS has been previously established (Sanders-Pinheiro et al., 2021; Marsicano et al., 2013; Elias and Cherukuri, 2023; Dobbels et al., 2010a).

#### 2.3.2 Multilevel correlates of immunosuppressive nonadherence

With Bronfenbrenner’s ecological model as a guide, variables that are predominantly at the patient level and include the micro level (healthcare providers/families), the meso level (healthcare organizations), and the macro level (healthcare systems and related policies) were investigated. The multilevel correlates of nonadherence were as follows: At the patient-level, factors included were sociodemographic factors, clinical factors, and treatment-related factors. Sociodemographic factors included age, gender, height, weight, body mass index (BMI), educational level (middle school and below, high school, and college), employment status (yes or no), marital status (steady partner or not), and family income (average disposable income, <¥20,000/year, ¥20,000–40,000/year, ¥40,000–80,000/year, and >¥80,000/year). Clinical factors consisted of chronic kidney disease etiology (polycystic kidney disease, nephrolith, chronic nephritis/nephropathy, others, unknown), time on the pre-KT treatment (years), pre-KT treatment modality (preemptive, peritoneal, hemodialysis, and peritoneal and hemodialysis). Treatment-related factors included post-transplant years, donor type (deceased or living donor), frequency of immunosuppressant regimens (1 or 2 doses/d), Tac IPV, adverse event episodes (self-reported medication side effects, yes/no). At the micro-level, the patients evaluated their satisfaction with the transplant team (good, fair, or poor). At the meso-level, the city of origin (same city, same province, or other provinces as the transplant center) was compiled. At the macro-level, the type of medical insurance (NRCMS, New Rural Cooperative Medical Scheme; UEBMI, Urban Employee Basic Medical Insurance, URBMI, Urban Resident Basic Medical Insurance) was documented.

#### 2.3.3 Data collection and assessments

Kidney transplant recipients who met the inclusion and exclusion criteria and signed the written consent form were interviewed by the transplantation team. Correlates were assessed by interviewers or were collected from the medical files. CV was used to quantify TAC IPV (Leino et al., 2019; Shuker et al., 2015b; Gonzales et al., 2020; Rahamimov et al., 2019). CV is expressed as the ratio of the standard deviation (σ) to the mean (μ). Using the time of adherence assessment as the follow-up baseline, ten TAC trough concentration levels were collected at the follow-up baseline and thereafter, and a minimum of three samples were required in the calculation of TAC IPV in renal transplant recipients. Renal function was assessed by serum creatinine value (SCr) and estimated glomerular filtration rate (eGFR). SCr was collected at baseline and at 3, 6, 9, and 12 months of follow-up, and eGFR was calculated according to the MDRD (the Modification of Diet in Renal Disease) formula. Banff 2017 was used to grade the biopsy specimens.

### 2.4 Statistical analysis

Data are expressed as mean ± standard deviation for normally distributed variables, median (interquartile range) for non-normally distributed variables, and number (proportion) for categorical variables. Factors potentially associated with adherence profiles were explored by comparison between latent classes, using the *t*-test and Mann–Whitney *U*-test for normally and non-normally distributed data, respectively, and the χ2 test or Fisher’s exact test for categorical data. We first considered nonadherence to immunosuppressants as a binary variable and performed univariate binary logistic regression analyses using generalized estimating equations assuming Binomial family and logit link function, providing original odds ratios (ORs) as associate parameters. Multivariate binary logistic regression analysis was performed for variables with *p* < 0.1. Repeated measures information was analyzed using repeated measures ANOVA. All statistical analyses were performed using the SPSS software version 26.0. A *p*-value < 0.05 was considered statistically significant.

## 3 Results

### 3.1 Sample characteristics

Between June 2022 and June 2023, 431 renal transplant recipients undergoing outpatient follow-up received adherence questionnaires, and 409 (94.9%) met the inclusion criteria of the analysis. There were 301 (73.6%) males and 108 (26.4%) females. The mean age of these patients was 39.6 ± 11.1 years, the mean BMI was 21.5 ± 3.4 kg/m^2^, and the median post-transplant period was 1.6 (IQR 3.3) years. A total of 317 (77.5%) recipients received kidneys from deceased donors while 92 (22.5%) received kidneys from living donors.

### 3.2 Prevalence of nonadherence to immunosuppressant medication

The present study showed a 41.6% (170/409) nonadherence rate to immunosuppressant medication in kidney transplant recipients. The most common issue of the four examined was taking nonadherence (forgetting), which was reported by 30.6% of patients, followed by timing nonadherence (delay >2 h) at 23%; whereas drug holidays (5.9%) and dose reduction (7.1%) were less frequently reported (Table 1).

**TABLE 1:** Medication adherence in kidney transplant recipients.

	Never	Once a month	Every 2 weeks	Every week	More than once a week	Every day
Taking nonadherence (forgetting) n (%)	284 (69.4)	73 (17.8)	42 (10.3)	5 (1.2)	4 (1.0)	1 (0.2)
Drug holidays n (%)	385 (94.1)	7 (1.7)	14 (3.4)	1 (0.2)	2 (0.5)	0 (0)
Timing nonadherence (delay >2 h) n (%)	315 (77)	46 (11.2)	34 (8.3)	7 (1.7)	7 (1.7)	0 (0)
Dose reduction n (%)	380 (92.9)	18 (4.4)	7 (1.7)	0 (0)	4 (1.0)	0 (0)

### 3.3 Multilevel correlates of immunosuppressant nonadherence

Descriptive statistics of the multilevel variables (implementation phase) and the results of the bivariate analysis for the two groups are shown in Table 2. Variables with *p* < 0.1, including educational level, marital status, post-transplant years, donor type, frequency of immunosuppressant regimens, and adverse event episodes, were included in the multiple logistic regression analysis. The results showed that two variables were positively associated with immunosuppressive nonadherence, namely, post-transplant years (OR: 1.240, 95% CI: 1.136–1.353, *p* < 0.001) and twice-daily immunosuppressive regimen (OR: 5.145, 95% CI: 2.690–9.840, *p* < 0.001). The risk of nonadherence in kidney transplant recipients increased by 24% for each additional year post-transplant. Recipients on twice-daily immunosuppression had a 5.145 times greater risk of nonadherence than recipients on single daily oral immunosuppression (Table 3).

**TABLE 2:** Descriptive statistics of the multilevel variables [adherent/nonadherent (implementation phase)] and results of bivariate analysis.

	Adherent (n = 239)	Nonadherent (n = 170)	*Odds ratio (95% CI), p*-value
Patient-level: sociodemographic, clinical, treatment-related factors			
Sociodemographic factors			
Age (year)	39.8 ± 11.1	39.3 ± 11.1	0.996 (0.978–1.014), 0.647
Male recipient n (%)	171 (71.5)	130 (76.5)	Reference
Female recipient n (%)	68 (28.5)	40 (23.5)	0.774 (0.492–1.216), 0.266
Height (cm)	168.5 ± 7.3	167.9 ± 7.8	0.989 (0.963–1.015), 0.407
Weight (kg)	60.8 ± 11.8	61.8 ± 11.4	1.008 (0.991–1.025), 0.382
Body mass index (kg/m^2^)	21.3 ± 3.5	21.8 ± 3.2	1.046 (0.987–1.109), 0.129
Educational level n (%)			
Middle school and below	43 (18)	44 (25.9)	Reference
High school	94 (39.3)	60 (35.3)	0.624 (0.367–1.060), 0.081
College	102 (42.7)	66 (38.8)	0.632 (0.375–1.066), 0.085
Employment status (%)			
Actively employed	125 (52.3)	85 (50.0)	Reference
Not actively employed	114 (47.7)	85 (50.0)	1.096 (0.740–1.625), 0.646
Marital status n (%)			
Steady partner	188 (78.7)	120 (70.6)	Reference
Without steady partner	51 (21.3)	50 (29.4)	1.536 (0.977–2.414), 0.063
Familiar income n (%)			
<¥20,000/year	56 (23.4)	49 (28.8)	Reference
¥20,000–40,000/year	78 (32.6)	57 (33.5)	0.835 (0.500–1.396), 0.492
¥40,000–80,000/year	57 (23.8)	34 (20.0)	0.682 (0.385–1.208), 0.189
>¥80,000/year	48 (20.1)	30 (17.6)	0.714 (0.394–1.296), 0.268
Clinical factors			
Chronic kidney disease etiology n (%)			
Polycystic kidney disease	3 (1.3)	3 (1.8)	Reference
Nephrolith	7 (2.9)	2 (1.2)	0.286 (0.030–2.692), 0.274
Chronic nephritis/nephropathy	133 (55.6)	88 (51.8)	0.662 (0.131–3.353), 0.662
Others	3 (1.3)	1 (0.6)	0.333 (0.021–5.329), 0.437
Unknown	93 (38.9)	76 (44.7)	0.817 (0.160–4.166), 0.808
Time on the pre-KT treatment n (%)			
<1 year	111 (46.4)	91 (53.5)	Reference
1–3 years	90 (37.7)	55 (32.4)	0.745 (0.482–1.152), 0.186
>3 years	38 (15.9)	24 (14.1)	0.770 (0.431–1.378), 0.379
Pre-KT treatment modality n (%)			
Preemptive	10 (4.2)	4 (2.4)	Reference
Peritoneal dialysis	27 (11.3)	19 (11.2)	1.759 (0.480–6.453), 0.394
Hemodialysis	195 (81.6)	143 (84.1)	1.833 (0.564–5.963), 0.314
Peritoneal & Hemodialysis	7 (2.9)	4 (2.4)	1.429 (0.264–7.737), 0.679
Treatment-related factors			
Post-transplant years^a^(IQR)	1.0 (2.4)	3.0 (4.4)	1.239 (1.142–1.345), <0.001
Type of donor n (%)			
Deceased donor	193 (80.8)	124 (72.9)	Reference
Living donor	46 (19.2)	46 (27.1)	1.556 (0.976–2.482), 0.063
Frequency of immunosuppressive regimens n (%)			
1 dose/d (QD)	77 (32.2)	13 (7.6)	Reference
2 doses/d (Q12)	162 (67.8)	157 (92.4)	5.740 (3.065–10.749), <0.001
Adverse event episodes n (%)	91 (38.1)	84 (49.4)	1.589 (1.067–2.366), 0.023
Micro-level: family/healthcare provider			
Patient satisfaction with the transplant team n (%)			
Good	227 (95)	155 (91.2)	Reference
Fair	11 (4.6)	13 (7.6)	1.731 (0.756–3.963), 0.194
Poor	1 (0.4)	2 (1.2)	2.929 (0.263–32.583), 0.382
Meso level: transplant center			
City of origin n (%)			
Other provinces of transplant center	5 (2.1)	4 (2.4)	Reference
Same province of transplant center	168 (70.3)	111 (65.3)	0.826 (0.217–3.143), 0.779
Same city of transplant center	66 (27.6)	55 (32.4)	1.042 (0.267–4.069), 0.953
Macro-level: healthcare system			
Type of medical insurance n (%)			
NRCMS	34 (14.2)	26 (15.3)	Reference
UEBMI	77 (32.3)	57 (33.5)	0.968 (0.523–1.790), 0.918
URBMI	128 (53.6)	87 (51.2)	0.889 (0.498–1.585), 0.690

^a^ Median (interquartile range) for non-normally distributed variables.

NRCMS, new rural cooperative medical scheme; UEBMI, urban employee basic medical insurance; URBMI, urban resident basic medical insurance.

**TABLE 3:** Multiple logistic regression analysis of nonadherence to immunosuppressives.

Nonadherence to immunosuppressives (BAASIS 4 questions)	Odds ratio	95% confidence interval	*p-value*
Educational level (High school)	0.839	0.478–1.528	0.558
Educational level (College)	0.854	0.478–1.528	0.596
Marital status (Without steady partner)	1.623	0.981–2.684	0.059
Post-transplant years	1.240	1.136–1.353	<0.001
Type of donor (Living donor)	1.021	0.607–1.717	0.937
Frequency of immunosuppressive regimens (2 doses/d)	5.145	2.690–9.840	<0.001
Adverse event episodes	1.482	0.951–2.311	0.082

### 3.4 Differences in IPV between adherent and nonadherent groups

**FIGURE 1:**
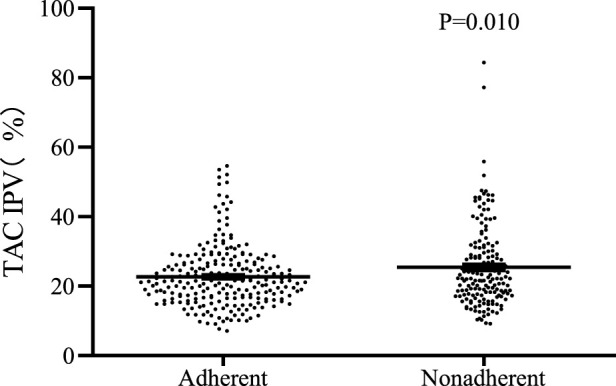
Distribution of TAC IPV in kidney transplant recipients in both groups.

A significant difference was observed in Tac IPV between the adherent and nonadherent groups (22.7 ± 8.7 vs. 25.4 ± 11.6, *p* = 0.010). There was no statistically significant difference in TAC IPV in the adherent and nonadherent groups, respectively, compared with the total sample (23.8 ± 10.1) (*p* = 0.147, *p* = 0.098, respectively) (Table 4) (Figure 1).

**TABLE 4:** Intra-patient variability of exposure to immunosuppressive drugs.

Tac trough concentration	Population	Mean (ng/mL)	SD (ng/mL)	Coefficient of variation (%)	Sample size (n)	*p-value*
Tacrolimus	Total sample	6.3 ± 1.7	1.5 ± 0.9	23.8 ± 10.1	409	0.147^a^/0.098^b^
	Adherents	6.7 ± 1.5	1.6 ± 0.9	22.7 ± 8.7	239	0.010
	Non-adherence	5.7 ± 1.9	1.4 ± 0.8	25.4 ± 11.6	170	

^a^ Adherents VS, total sample.

^b^ Non-adherence VS, total sample.

^c^ Adherents VS, Non-adherence.

### 3.5 Graft renal function

**FIGURE 2:**
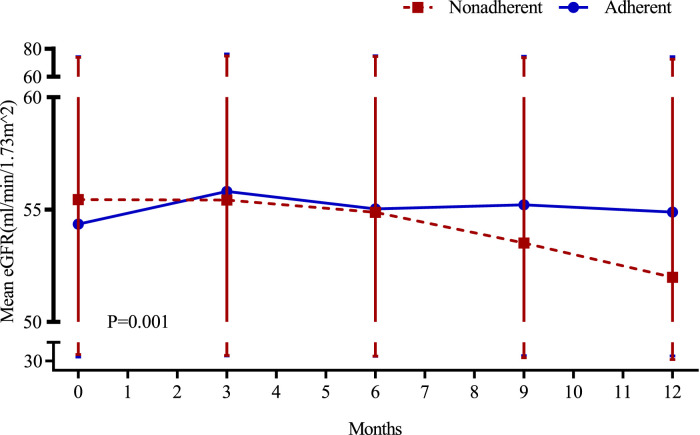
Variation in renal function in both groups of recipients.

Repeated-measures ANOVA showed a time-dependent interaction between mean eGFR (F = 4.932, *p* = 0.001) in the two groups, indicating that the difference in mean eGFR fluctuations between the two groups was statistically significant over time. The results of *post hoc* multiple comparisons analysis showed that the mean eGFR of the recipients in the adherence group remained stable during the follow-up period (follow-up baseline: 54.35 ± 19.97 mL/min/1.73 m2, follow-up 3 months: 55.81 ± 20.33 mL/min/1.73 m2, follow-up 6 months: 55.03 ± 19.97 mL/min/1.73 m2, follow-up 9 months: 55.22 ± 19.42 mL/min/1.73 m2, 12 months of follow-up: 54.89 ± 19.31 mL/min/1.73 m2), the difference between mean eGFR at 3 months of follow-up and mean eGFR at baseline of follow-up was statistically significant only (*p* = 0.001). In contrast, recipients in the nonadherence group showed a decreasing trend in mean eGFR during the follow-up period (follow-up baseline: 55.44 ± 18.39 mL/min/1.73 m2, follow-up 3 months: 55.43 ± 19.36 mL/min/1.73 m2, follow-up 6 months: 54.88 ± 19.53 mL/min/1.73 m2, follow-up 9 months: 53.51 ± 20.08 mL/min/1.73 m2, follow-up 12 months: 51.99 ± 20.49 mL/min/1.73 m2), and the difference between the mean eGFR at follow-up 9 months and follow-up 12 months and the mean eGFR at follow-up baseline was statistically significant (*p* = 0.009, *p* < 0.001, respectively). The curves of mean eGFR changes during the follow-up period between the two groups are shown in Figure 2.

### 3.6 Clinical outcomes

**FIGURE 3:**
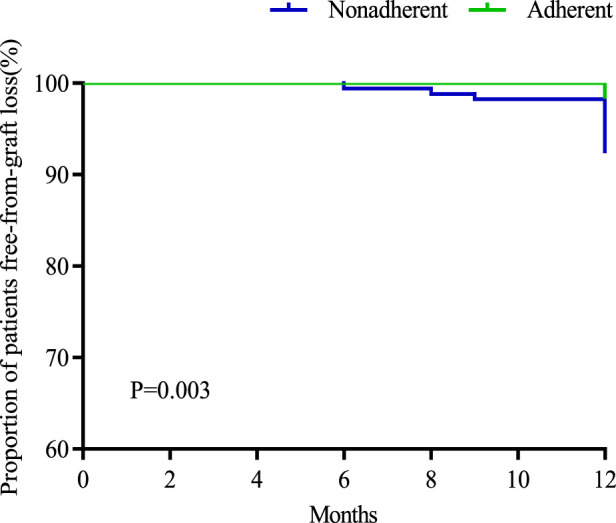
Survival curves for recipients without graft loss in both groups.

Among 409 renal transplant recipients, 16 cases of graft loss, 2 cases of recipient death, and 31 cases of rejection, the rates of graft loss, recipient mortality, and rejection were 3.9%, 0.5%, and 7.6%, respectively. The incidence of graft loss and recipient death was analyzed and compared between the two groups. The results showed that there were no recipient deaths in the adherence group, and 2 recipient deaths in the non-adherence group, with the causes of death being pulmonary infection and cerebral hemorrhage, respectively, and the difference in the incidence of recipient deaths between the two groups was not statistically significant (*p* = 0.336). The incidence of graft loss was 1.7% (4/239) in the adherence group and 7.1% (12/170) in the non-adherence group, and the incidence of graft loss was higher in the non-adherence group than in the adherence group and the difference was statistically significant (*p* = 0.006) (Table 5) (Figure 3).

**TABLE 5:** Comparison of the incidence of rejection episodes between the two groups.

	Adherent (n = 239)	Nonadherent (n = 170)	*p-value*
Death n (%)	0 (0)	2 (1.2)	0.336
Graft loss n (%)	4 (1.7)	12 (7.1)	0.006
Rejection episodes n (%)	10 (4.2)	21 (12.4)	0.002
Type of rejection episodes			
Cellular n (%)	8 (3.3)	9 (5.3)	0.331
Humoral n (%)	1 (0.4)	8 (4.7)	0.010
Combined n (%)	1 (0.4)	4 (2.4)	0.194

**FIGURE 4:**
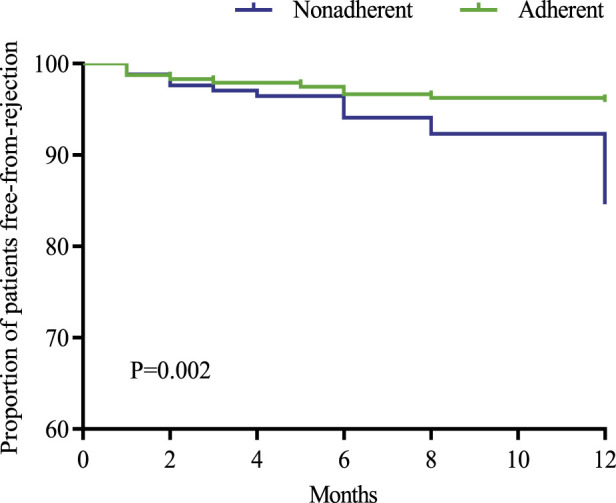
Survival curves for recipients without rejection in both groups.

The incidence of rejection in the two groups was analyzed and compared. The incidence of rejection was 4.2% (10/239) in the adherent group and 12.4% (21/170) in the nonadherent group, and the incidence of rejection in the nonadherent group was higher than that in the adherent group and the difference was statistically significant (*p* = 0.002). In terms of different types of rejection, the incidence of antibody-mediated rejection was higher in the non-adherence group than in the adherence group and the difference was statistically significant (4.7% vs. 0.4%, *p* = 0.01) (Table 5) (Figure 4).

## 4 Discussion

This study assessed the prevalence of nonadherence to immunosuppressant therapy (implementation phase) in Chinese renal transplant recipients and explored the multilevel correlates associated with nonadherence. In addition, we investigated the relationship between Tac IPV and nonadherence, as well as the relationship between nonadherence and clinical outcomes.

We assessed medication adherence in kidney transplant recipients using BAASIS, which has good validity and reliability as a self-report tool for assessing medication nonadherence in transplant recipients and can be easily implemented clinically. Our results showed a high prevalence of nonadherence to immunosuppressant therapy in renal transplant recipients of 41.6%, and the most common issue of the four examined was taking nonadherence (forgetting) (30.6%). Some studies that also used BAASIS to report nonadherence showed similar results in adult (Dew et al., 2007; Pabst et al., 2015; Lennerling and Forsberg, 2012; Scheel et al., 2017) and pediatric (Dobbels et al., 2010b) renal transplant recipients (30%–54%). The ADHERE BRAZIL showed an overall prevalence of immunosuppressant nonadherence in kidney transplant recipients of 39.7% (range 11.0%–65.2%). The highest prevalence of the four independent dimensions of BAASIS was medication timing deviation (30.6%), followed by not taking immunosuppressants on time (14.3%) (Sanders-Pinheiro et al., 2021). While Meskerem Nimani Derejie et al. showed a 23.0% probability of immunosuppression nonadherence in renal transplant recipients, with 60.7% of recipients citing forgetfulness as the reason for nonadherence to medication (Derejie et al., 2024). However, that these data may be subject to recruitment bias and/or social desirability needs to be considered (Scheel et al., 2017).

The potential correlates of the four levels of nonadherence based on the Ecological Model were investigated in our study. The assessment of patient-level factors was more frequent, whereas that of micro-, meso-, and macro-level factors was relatively lacking. At the patient level, our study showed that lack of a steady partner, longer post-transplantation time, higher Tac IPV, and a higher frequency of immunosuppressant medication were positively associated with nonadherence. These results are consistent with previous studies in which longer post-transplant time was a risk factor for nonadherence, with an increasing proportion of nonadherent recipients over time (Gokoel et al., 2020; Nerini et al., 2016; Belaiche et al., 2017). Several interventional and observational studies have confirmed that reducing pill load and decreasing medication frequency can significantly improve adherence (van Zanten et al., 2021; Kobayashi et al., 2020; Oh et al., 2020). Although extended-release Tac (ER-Tac) allows once-daily dosing, which has the potential to improve treatment adherence, mycophenolate acid (MPA) does not allow once-daily dosing; therefore, the current mainstream immunosuppressive regimen, TAC/MPA/prednisone, still requires twice-daily dosing. In contrast to previous studies, we achieved a true once-daily immunosuppressive regimen by using sirolimus (SRL) in combination with low-dose ER-TAC, and renal transplant recipients receiving this simplified once-daily immunosuppressive regimen showed significantly improved medication adherence. Some of the classical correlates associated with nonadherence, such as age (Kidney Disease: Improving Global Outcomes KDIGO Transplant Work Group, 2009; Nerini et al., 2016; Belaiche et al., 2017; Spivey et al., 2014; Kindem et al., 2023; Varnell et al., 2022), male sex (Kobayashi et al., 2020), and socioeconomic level nonadherence (Gokoel et al., 2020; Nerini et al., 2016; Kobayashi et al., 2020), were not detected in our study. The ADHERE BRAZIL reveals patient level-having a stable partner (OR: 0.75; CI: 0.58–0.97), nonadherence to appointments (OR: 2.98; CI: 2.03–4.39), and nonadherence to physical activity recommendations (OR: 1.84; CI: 1.38–2.46); and transplant center level-satisfaction with the waiting room structure (OR: 0.54; CI: 0.42–0.71), consultation >30 min (OR: 1.60; CI: 1.19–2.14), adequacy of the consultation frequency (OR: 0.62; CI: 0.43–0.90), and centers with >500 beds (OR: 0.58; CI: 0.46–0.73) were independently associated with nonadherence (Sanders-Pinheiro et al., 2021). However, because this study was a single-center study, exploration was limited of the other three levels of correlation. We did not find a significant association between patient satisfaction with the transplant team (micro-level), city of origin (meso-level), or type of medical insurance (macro-level) with nonadherence to treatment. China has committed to universal health coverage and “Healthy China 2030”. Since this healthcare reform, out-of-pocket expenditures as a percentage of the current health expenditures in China have dropped dramatically. Health insurance reform has been achieved in terms of the breadth of coverage in the population, the comprehensiveness of the benefits packages, and increased reimbursement rates. The coverage of healthcare services has progressed greatly in terms of accessibility, equity, and quality (Fang et al., 2019; Tao et al., 2020; Zhang et al., 2020; Liu et al., 2021). However, subsequent expanded studies with more comprehensive multilevel correlations are needed.

We calculated the Tac IPV using CV and explored the relationship between Tac IPV and medication adherence. The results showed that the mean Tac IPV was 23.9%, which was similar to previous studies (Schumacher et al., 2021; Kostalova et al., 2022). A correlation between Tac IPV and self-reported medication adherence was also found, and recipients with high IPV were more likely to experience nonadherence. Therefore, Tac IPV may serve as a predictor of adherence. A review of Tac IPV suggested that Tac IPV could be used as a surrogate marker of adherence, and there have been studies that have used questionnaires combined with IPV to assess adherence (Gonzales et al., 2020; Mella et al., 2023). But some studies showed no significant correlation between Tac IPV and adherence (Ko et al., 2021). Although there is no gold standard for clinical adherence monitoring, and different methods of adherence monitoring have different variabilities, Tac IPV can be used as an additional tool to identify recipients at risk of nonadherence (Kostalova et al., 2022).

The results of the present study showed that over time, renal function remained stable during the follow-up period in the adherent group, whereas there was a gradual decline in renal function during the follow-up period in the nonadherent group. The difference in renal function between the two transplantation groups may be related to the higher incidence of rejection and graft loss in the nonadherence group, which also confirms that nonadherence may be a risk factor for renal function in transplantation. The results of MAGIC, a comprehensive clinical trial examining the SystemCHANGE™ intervention in improving adherence to immunosuppressive medications and improving prognosis in adult renal transplant recipients, showed that adherence was significantly better in the intervened recipients than in the non-intervened recipients, and that the mean serum creatinine values and urea nitrogen were lower in the adherent recipients at the 12th month of follow-up (Russell et al., 2020).

This study investigated the effect of immunosuppressant nonadherence on graft outcome and rejection in renal transplant recipients. The results of the study showed no significant difference in mortality of recipients in both groups (*p* = 0.336), while the incidence of graft loss was higher in the nonadherence group than in the adherence group (7.1% vs. 1.7%, *p* = 0.006). The incidence of rejection in recipients in the non-adherence group was also higher than that in the adherence group and the difference was statistically significant (12.4% vs. 7.6%, *p* = 0.002), especially for antibody-mediated rejection (4.7% vs. 0.4%, *p* = 0.01). Several foreign studies on adherence and transplantation outcomes have shown that improved adherence improves transplantation outcomes, including heart transplantation, liver transplantation, lung transplantation, and kidney transplantation (Russell et al., 2020; Dobbels et al., 2017). It was reported that immunosuppressant nonadherence was an independent correlate of late rejection and dnDSA, and interstitial fibrosis and tubular atrophy found in late post-transplant biopsies were associated with early TCMR and immunosuppressant nonadherence. Allogeneic immune-mediated late graft loss ensues as a result of persistent AMR and/or TCMR, both of which may be accelerated by recipient immunosuppression nonadherence or minimal immunosuppression (Nevins et al., 2017). A study of interventions to improve adherence showed that implementation of the intervention reduced the incidence of rejection by 50% (RR: 0.50, 95% CI: 0.27–0.91, *p* = 0.02) [80]. In addition, studies on the impact of TAC IPV on clinical outcomes in renal transplantation have reported that high TAC IPV adversely affects graft survival, acute rejection, dnDSA, chronic immune-mediated graft injury, and histologic lesions (Gonzales et al., 2020). It is particularly important to identify risk factors for nonadherence to immunosuppressive medications and implement appropriate interventions to improve medication adherence in recipients. Due to the special characteristics of renal transplant recipients as chronic disease patients and the need for lifelong oral administration and monitoring of immunosuppressive drugs, medication adherence is more important for renal transplant recipients than for other chronic disease patients, and adherence is an indicator of long-term concern for renal transplant recipients. The European COMMIT consensus suggests that adherence should be considered as the fifth vital sign of concern in renal transplant recipients (Neuberger et al., 2017), assessed at each follow-up visit, and the long-term impact of adherence on renal transplant recipients requires further extended research.

Our study has several limitations. First, the multilevel correlates of nonadherence, especially micro-, meso-, and macro-level correlates, were not explored comprehensively, and further multicenter prospective studies are warranted to explore the multilevel correlates of nonadherence in a more comprehensive manner. Second, a validated self-report survey was used to assess adherence, and this measure of adherence may overestimate or underestimate nonadherence because of the subjective nature of the recipients. We did not use more objective reference methods for nonadherence, such as electronic medication monitoring systems. These methods are considered the closest to the gold standard for measuring nonadherence, but they are both expensive and difficult to implement in routine clinical treatment. Third, we measured IPV only for Tac, ignoring the effects of other immunosuppressive agents (including cyclosporine and mammalian target of rapamycin [mTOR] inhibitors). Although each solid-organ transplant recipient is subjected to regular therapeutic drug monitoring with calcineurin inhibitors (cyclosporine and tacrolimus) and/or mTOR inhibitors (sirolimus and everolimus) during post-transplant follow-up, the most common assessment of immunosuppression nonadherence by therapeutic drug monitoring is the variability of Tac trough levels. Moreover, our study lacked risk factors associated with Tac IPV, such as age, sex, BMI, genetic polymorphisms in *CYP3A5* and *CYP3A4*, drug interactions, liver function, and lifestyle choices account for the differences in IPV. Similarly, IPV is affected by adherence, gastrointestinal metabolism and motility, diarrhea, food and drug interactions, synchronicity of dose administration and blood tests, and variability in the laboratory assays. Therefore, deeper exploration is needed to further substantiate the relationship between Tac IPV and nonadherence. In addition, further studies are needed to investigate the relationship between nonadherence and IPV of immunosuppressive agents with long-term renal transplant outcomes, and to explore further prospective interventions for modifiable risk factors associated with nonadherence.

In conclusion, the incidence of nonadherence to immunosuppressant therapy in renal transplant recipients in this study was as high as 41.6%. Longer time since the transplantation and higher frequency of immunosuppressive dosing were positively associated with nonadherence to immunosuppressives medication. Immunosuppressant nonadherence was associated with adverse graft outcomes.

## Data Availability

The raw data supporting the conclusions of this article will be made available by the authors, without undue reservation.

## References

[B1] Basel assessment of adherence to immunosuppressive medications scale (BAASIS©) – EXPLANATION (2024). Available at: https://baasis.nursing.unibas.ch/data/2020-BAASIS-explanation.pdf.

[B3] Belaiche S. Décaudin B. Dharancy S. Noel C. Odou P. Hazzan M. (2017). Factors relevant to medication non-adherence in kidney transplant: a systematic review. Int. J. Clin. Pharm. 39 (3), 582–593. 10.1007/s11096-017-0436-4 28374343

[B4] Berben L. Dobbels F. Engberg S. Hill M. N. De Geest S. (2012). An ecological perspective on medication adherence. J. Nurs. Res. 34 (5), 635–653. 10.1177/0193945911434518 22309989

[B5] Burkhart P. V. Sabaté E. (2003). Adherence to long-term therapies: evidence for action. J. Nurs. Scholarsh. 35 (3), 207. 10.1111/j.1547-5069.2003.tb00001.x 14562485

[B6] Chen T. Wang Y. Tian D. Zhang J. Xu Q. Lv Q. (2022). Follow-up factors contribute to immunosuppressant adherence in kidney transplant recipients. Patient Prefer Adherence 16, 2811–2819. 10.2147/PPA.S383243 36284546 PMC9588292

[B7] Cherukuri A. Mehta R. Sharma A. Sood P. Zeevi A. Tevar A. D. (2019). Post-transplant donor specific antibody is associated with poor kidney transplant outcomes only when combined with both T-cell-mediated rejection and non-adherence. Kidney Int. 96 (1), 202–213. 10.1016/j.kint.2019.01.033 31029504

[B8] Denhaerynck K. Berben L. Dobbels F. Russell C. L. Crespo-Leiro M. G. Poncelet A. J. (2018). Multilevel factors are associated with immunosuppressant nonadherence in heart transplant recipients: the international BRIGHT study. Am. J. Transpl. 18 (6), 1447–1460. 10.1111/ajt.14611 PMC6001479 29205855

[B9] Denhaerynck K. Dobbels F. Košťálová B. De Geest S. ASIS Consortium (2023). Psychometric properties of the BAASIS: a meta-analysis of individual participant data. Transplantation 107 (8), 1795–1809. 10.1097/TP.0000000000004574 36949037 PMC10358438

[B10] Derejie M. N. Dereje E. N. Alemu D. M. Tesfay Y. G. Hunduma F. Temie N. M. (2024). Medication non-adherence and its associated factors among kidney transplant patients in a large teaching hospital in Ethiopia. BMC Nephrol. 25 (1), 187. 10.1186/s12882-024-03620-z 38824513 PMC11144307

[B11] Dew M. A. DiMartini A. F. De Vito Dabbs A. Myaskovsky L. Steel J. Unruh M. (2007). Rates and risk factors for nonadherence to the medical regimen after adult solid organ transplantation. Transplantation 83 (7), 858–873. 10.1097/01.tp.0000258599.65257.a6 17460556

[B12] Dobbels F. Berben L. De Geest S. Drent G. Lennerling A. Whittaker C. (2010a). The psychometric properties and practicability of self-report instruments to identify medication nonadherence in adult transplant patients: a systematic review. Transplantation 90 (2), 205–219. 10.1097/TP.0b013e3181e346cd 20531073

[B13] Dobbels F. De Bleser L. Berben L. Kristanto P. Dupont L. Nevens F. (2017). Efficacy of a medication adherence enhancing intervention in transplantation: the MAESTRO-Tx trial. J. Heart Lung Transpl. 36 (5), 499–508. 10.1016/j.healun.2017.01.007 28162931

[B14] Dobbels F. Ruppar T. De Geest S. Decorte A. Van Damme-Lombaerts R. Fine R. N. (2010b). Adherence to the immunosuppressive regimen in pediatric kidney transplant recipients: a systematic review. Pediatr. Transplant. 14 (5), 603–613. 10.1111/j.1399-3046.2010.01299.x 20214741

[B15] Eisenberger U. Wüthrich R. P. Bock A. Ambühl P. Steiger J. Intondi A. (2013). Medication adherence assessment: high accuracy of the new Ingestible Sensor System in kidney transplants. Transplantation 96 (3), 245–250. 10.1097/TP.0b013e31829b7571 23823651 PMC3749815

[B16] Elias C. Cherukuri A. (2023). BAASIS for monitoring therapy nonadherence in clinical transplantation: are we there yet? Transplantation 107, 1673–1674. 10.1097/TP.0000000000004575 36949031 PMC10363179

[B17] Fang H. Eggleston K. Hanson K. Wu M. (2019). Enhancing financial protection under China's social health insurance to achieve universal health coverage. Bmj 365, l2378. 10.1136/bmj.l2378 31227485 PMC6598720

[B18] Gandolfini I. Palmisano A. Fiaccadori E. Cravedi P. Maggiore U. (2022). Detecting, preventing and treating non-adherence to immunosuppression after kidney transplantation. Clin. Kidney J. 15 (7), 1253–1274. 10.1093/ckj/sfac017 35756738 PMC9217626

[B19] Gokoel S. R. M. Gombert-Handoko K. B. Zwart T. C. van der Boog P. J. M. Moes D. de Fijter J. W. (2020). Medication non-adherence after kidney transplantation: a critical appraisal and systematic review. Transpl. Rev. Orl. 34 (1), 100511. 10.1016/j.trre.2019.100511 31627978

[B20] Gonzales H. M. McGillicuddy J. W. Rohan V. Chandler J. L. Nadig S. N. Dubay D. A. (2020). A comprehensive review of the impact of tacrolimus intrapatient variability on clinical outcomes in kidney transplantation. Am. J. Transpl. 20 (8), 1969–1983. 10.1111/ajt.16002 PMC11140479 32406604

[B21] Gustavsen M. T. Midtvedt K. Lønning K. Jacobsen T. Reisaeter A. V. De Geest S. (2019). Evaluation of tools for annual capture of adherence to immunosuppressive medications after renal transplantation - a single-centre open prospective trial. Transpl. Int. official J. Eur. Soc. Organ Transplant. 32 (6), 614–625. 10.1111/tri.13412 30770608

[B22] Kidney Disease: Improving Global Outcomes KDIGO Transplant Work Group (2009). KDIGO clinical practice guideline for the care of kidney transplant recipients. Am. J. Transpl. 9 (Suppl. 3), S1–S155. 10.1111/j.1600-6143.2009.02834.x 19845597

[B23] Kindem I. A. Bjerre A. Hammarstrøm C. Naper C. Midtvedt K. Åsberg A. (2023). Kidney-transplanted adolescents-nonadherence and graft outcomes during the transition phase: a nationwide analysis, 2000-2020. Transplantation 107 (5), 1206–1212. 10.1097/TP.0000000000004431 36476728 PMC10125107

[B24] Ko H. Kim H. K. Chung C. Han A. Min S. K. Ha J. (2021). Association between medication adherence and intrapatient variability in tacrolimus concentration among stable kidney transplant recipients. Sci. Rep. 11 (1), 5397. 10.1038/s41598-021-84868-5 33686160 PMC7940492

[B25] Kobayashi S. Tsutsui J. Okabe S. Hideki I. Akaho R. Nishimura K. (2020). Medication nonadherence after kidney transplantation: an internet-based survey in Japan. Psychol. Health Med. 25 (1), 91–101. 10.1080/13548506.2019.1622745 31144516

[B26] Kostalova B. Mala-Ladova K. Sulkova S. D. Denhaerynck K. De Geest S. Maly J. (2022). Comparison of different methods to assess tacrolimus concentration intra-patient variability as potential marker of medication non-adherence. Front. Pharmacol. 13, 973564. 10.3389/fphar.2022.973564 36313323 PMC9609782

[B27] Lam W. Y. Fresco P. (2015). Medication adherence measures: an overview. Biomed. Res. Int. 2015, 217047. 10.1155/2015/217047 26539470 PMC4619779

[B28] Leino A. D. King E. C. Jiang W. Vinks A. A. Klawitter J. Christians U. (2019). Assessment of tacrolimus intrapatient variability in stable adherent transplant recipients: establishing baseline values. Am. J. Transpl. 19 (5), 1410–1420. 10.1111/ajt.15199 30506623

[B29] Lennerling A. Forsberg A. (2012). Self-reported non-adherence and beliefs about medication in a Swedish kidney transplant population. Open Nurs. J. 6, 41–46. 10.2174/1874434601206010041 22509233 PMC3322447

[B30] Lentine K. L. Smith J. M. Hart A. Miller J. Skeans M. A. Larkin L. (2022). OPTN/SRTR 2020 annual data report: kidney. Am. J. Transpl. 22 (Suppl. 2), 21–136. 10.1111/ajt.16982 35266618

[B31] Liu X. Wang Z. Zhang H. Meng Q. (2021). Measuring and evaluating progress towards universal health coverage in China. J. Glob. Health 11, 08005. 10.7189/jogh.11.08005 33981413 PMC8088770

[B32] Marsicano E. O. Fernandes Nda S. Colugnati F. Grincenkov F. R. Fernandes N. M. De Geest S. (2013). Transcultural adaptation and initial validation of Brazilian-Portuguese version of the Basel assessment of adherence to immunosuppressive medications scale (BAASIS) in kidney transplants. BMC Nephrol. 14, 108. 10.1186/1471-2369-14-108 23692889 PMC3665586

[B33] Marston M. T. Berben L. Dobbels F. Russell C. L. de Geest S. (2023). Prevalence and patient-level correlates of intentional non-adherence to immunosuppressive medication after heart-transplantation-findings from the international BRIGHT study. Transpl. Int. official J. Eur. Soc. Organ Transplant. 36, 11308. 10.3389/ti.2023.11308 PMC10363605 37492859

[B34] Mella A. Torazza M. C. Finocchietti D. Fop F. Allesina A. Dolla C. (2023). Non-adherence assessment to immunosuppressant therapy with a self-report questionnaire and intra-patient variability in renal transplantation: risk factors and clinical correlations. Minerva Urol. Nephrol. 75 (1), 92–98. 10.23736/S2724-6051.21.04244-2 33781021

[B35] Nerini E. Bruno F. Citterio F. Schena F. P. (2016). Nonadherence to immunosuppressive therapy in kidney transplant recipients: can technology help? J. Nephrol. 29 (5), 627–636. 10.1007/s40620-016-0273-x 26885659

[B36] Neuberger J. M. Bechstein W. O. Kuypers D. R. Burra P. Citterio F. De Geest S. (2017). Practical recommendations for long-term management of modifiable risks in kidney and liver transplant recipients: a guidance report and clinical checklist by the consensus on managing modifiable risk in transplantation (COMMIT) group. Transplantation 101 (4S Suppl. 2), S1-S56–s56. 10.1097/TP.0000000000001651 28328734

[B37] Nevins T. E. Nickerson P. W. Dew M. A. (2017). Understanding medication nonadherence after kidney transplant. J. Am. Soc. Nephrol. 28 (8), 2290–2301. 10.1681/ASN.2017020216 28630231 PMC5533244

[B38] Oh C. K. Bang J. B. Kim S. J. Huh K. H. Kim S. J. Jeon J. S. (2020). Improvement of medication adherence with simplified once-daily immunosuppressive regimen in stable kidney transplant recipients: a prospective cohort study. Asian J. Surg. 43 (6), 660–667. 10.1016/j.asjsur.2019.07.011 31353239

[B39] Pabst S. Bertram A. Zimmermann T. Schiffer M. de Zwaan M. (2015). Physician reported adherence to immunosuppressants in renal transplant patients: prevalence, agreement, and correlates. J. Psychosom. Res. 79 (5), 364–371. 10.1016/j.jpsychores.2015.09.001 26526310

[B40] Rahamimov R. Tifti-Orbach H. Zingerman B. Green H. Schneider S. Chagnac A. (2019). Reduction of exposure to tacrolimus trough level variability is associated with better graft survival after kidney transplantation. Eur. J. Clin. Pharmacol. 75 (7), 951–958. 10.1007/s00228-019-02643-y 30762079

[B41] Rodrigo E. Segundo D. S. Fernández-Fresnedo G. López-Hoyos M. Benito A. Ruiz J. C. (2016). Within-patient variability in tacrolimus blood levels predicts kidney graft loss and donor-specific antibody development. Transplantation 100 (11), 2479–2485. 10.1097/TP.0000000000001040 26703349

[B42] Russell C. L. Hathaway D. Remy L. M. Aholt D. Clark D. Miller C. (2020). Improving medication adherence and outcomes in adult kidney transplant patients using a personal systems approach: SystemCHANGE™ results of the MAGIC randomized clinical trial. Am. J. Transpl. 20 (1), 125–136. 10.1111/ajt.15528 PMC7179766 31291507

[B43] Sanders-Pinheiro H. Colugnati F. A. B. Denhaerynck K. Marsicano E. O. Medina J. O. P. De Geest S. (2021). Multilevel correlates of immunosuppressive nonadherence in kidney transplant patients: the multicenter adhere Brazil study. Transplantation 105 (1), 255–266. 10.1097/TP.0000000000003214 32150041

[B44] Sanders-Pinheiro H. Colugnati F. A. B. Marsicano E. O. De Geest S. Medina J. O. P. Adhere Brazil Consortium Group (2018). Prevalence and correlates of non-adherence to immunosuppressants and to health behaviours in patients after kidney transplantation in Brazil - the ADHERE Brazil multicentre study: a cross-sectional study protocol. BMC Nephrol. 19 (1), 41. 10.1186/s12882-018-0840-6 29463231 PMC5819659

[B45] Sapir-Pichhadze R. Wang Y. Famure O. Li Y. Kim S. J. (2014). Time-dependent variability in tacrolimus trough blood levels is a risk factor for late kidney transplant failure. Kidney Int. 85, 1404–1411. 10.1038/ki.2013.465 24336032

[B46] Schäfer-Keller P. Steiger J. Bock A. Denhaerynck K. De Geest S. (2008). Diagnostic accuracy of measurement methods to assess non-adherence to immunosuppressive drugs in kidney transplant recipients. Am. J. Transpl. 8 (3), 616–626. 10.1111/j.1600-6143.2007.02127.x 18294158

[B47] Scheel J. Reber S. Stoessel L. Waldmann E. Jank S. Eckardt K. U. (2017). Patient-reported non-adherence and immunosuppressant trough levels are associated with rejection after renal transplantation. BMC Nephrol. 18 (1), 107. 10.1186/s12882-017-0517-6 28356080 PMC5372303

[B48] Schönfeld S. Denhaerynck K. Berben L. Dobbels F. Russell C. L. Crespo-Leiro M. G. (2020). Prevalence and correlates of cost-related medication nonadherence to immunosuppressive drugs after heart transplantation: the international multicenter cross-sectional bright study. J. Cardiovasc Nurs. 35 (6), 519–529. 10.1097/JCN.0000000000000683 32433348 PMC7553198

[B49] Schumacher L. Leino A. D. Park J. M. (2021). Tacrolimus intrapatient variability in solid organ transplantation: a multiorgan perspective. Pharmacotherapy 41 (1), 103–118. 10.1002/phar.2480 33131078

[B50] Shemesh E. Bucuvalas J. C. Anand R. Mazariegos G. V. Alonso E. M. Venick R. S. (2017). The medication level variability index (MLVI) predicts poor liver transplant outcomes: a prospective multi-site study. Am. J. Transpl. 17 (10), 2668–2678. 10.1111/ajt.14276 PMC5607074 28321975

[B51] Shuker N. Gelder T. Hesselink D. A. (2015b). Intra-patient variability in tacrolimus exposure: causes, consequences for clinical management. Transpl. Rev. 9. 10.1016/j.trre.2015.01.002 25687818

[B52] Shuker N. van Gelder T. Hesselink D. A. (2015a). Intra-patient variability in tacrolimus exposure: causes, consequences for clinical management. Transpl. Rev. Orl. 29 (2), 78–84. 10.1016/j.trre.2015.01.002 25687818

[B53] Spivey C. A. Chisholm-Burns M. A. Damadzadeh B. Billheimer D. (2014). Determining the effect of immunosuppressant adherence on graft failure risk among renal transplant recipients. Clin. Transplant. 28 (1), 96–104. 10.1111/ctr.12283 24329814

[B54] Tao W. Zeng Z. Dang H. Li P. Chuong L. Yue D. (2020). Towards universal health coverage: achievements and challenges of 10 years of healthcare reform in China. BMJ Glob. Health 5 (3), e002087. 10.1136/bmjgh-2019-002087 PMC7103842 32257401

[B55] Teng S. Zhang S. Zhang W. Lin X. Shang Y. Peng X. (2015). Symptom experience associated with immunosuppressive medications in Chinese kidney transplant recipients. J. Nurs. Scholarsh. 47 (5), 425–434. 10.1111/jnu.12157 26219726

[B2] The Basel assessment of adherence to immunoSuppressive medIcations Scale© (2024). Available at: https://baasis.nursing.unibas.ch/.

[B56] van Zanten R. de Weerd A. Betjes M. Boer-Verschragen M. Massey E. K. (2021). Is simplification of immunosuppressive medication a way to promote medication adherence of kidney transplant recipients? Findings from a randomized controlled trial. Transpl. Int. official J. Eur. Soc. Organ Transplant. 34 (9), 1703–1711. 10.1111/tri.13993 PMC9292224 34448273

[B57] Varnell C. D. Jr. Rich K. L. Modi A. C. Hooper D. K. Eckman M. H. (2022). A cost-effectiveness analysis of adherence promotion strategies to improve rejection rates in adolescent kidney transplant recipients. Am. J. Kidney Dis. 80 (3), 330–340. 10.1053/j.ajkd.2021.12.013 35227823 PMC9398956

[B58] Villeneuve C. Rousseau A. Rerolle J. P. Couzi L. Kamar N. Essig M. (2020). Adherence profiles in kidney transplant patients: causes and consequences. Patient Educ. Couns. 103 (1), 189–198. 10.1016/j.pec.2019.08.002 31447197

[B59] Vrijens B. De Geest S. Hughes D. A. Przemyslaw K. Demonceau J. Ruppar T. (2012). A new taxonomy for describing and defining adherence to medications. Br. J. Clin. Pharmacol. 73 (5), 691–705. 10.1111/j.1365-2125.2012.04167.x 22486599 PMC3403197

[B60] Whalen H. R. Glen J. A. Harkins V. Stevens K. K. Jardine A. G. Geddes C. C. (2017). High intrapatient tacrolimus variability is associated with worse outcomes in renal transplantation using a low-dose tacrolimus immunosuppressive regime. Transplantation 101 (2), 430–436. 10.1097/TP.0000000000001129 26950724

[B61] Yang L. Liu H. X. Hu Y. Zhang B. Peng F. C. Wei C. Y. (2022). Exploration of adherence to the immunosuppressive medication in kidney transplant recipients based on theory of planned behavior. Clin. Nurs. Res. 31 (6), 1189–1198. 10.1177/10547738221096550 35575261

[B62] Zhang H. Zhang C. Zhu S. Ye H. Zhang D. (2020). Direct medical costs of end-stage kidney disease and renal replacement therapy: a cohort study in Guangzhou City, southern China. BMC Health Serv. Res. 20 (1), 122. 10.1186/s12913-020-4960-x 32059726 PMC7023821

[B63] Zhao L. Yan J. Yang G. L. Liu Y. (2017). A study on adherence to follow-up, quality of life, and associated factors among renal transplant recipients in China. Transplant. Proc. 49 (6), 1285–1290. 10.1016/j.transproceed.2017.03.086 28735995

